# Abnormal collagen deposition mediated by cartilage oligomeric matrix protein in the pathogenesis of oral submucous fibrosis

**DOI:** 10.1038/s41368-025-00355-x

**Published:** 2025-03-27

**Authors:** Yafei Xiong, Xuechun Li, Bincan Sun, Jie Zhang, Xiaoshan Wu, Feng Guo

**Affiliations:** 1 Department of Oral and Maxillofacial Surgery, Xiangya Hospital, Central South University, Changsha, China; 2 Academician Workstation for Oral-Maxillofacial Regenerative Medicine, Central South University, Changsha, China; 3 Research Center of Oral and Maxillofacial Development and Regeneration, Xiangya Hospital, Central South University, Changsha, China; 4 National Clinical Research Center for Geriatric Diseases, Xiangya Hospital, Central South University, Changsha, China

**Keywords:** Oral diseases, Biomarkers

## Abstract

Abnormal accumulation of collagen fibrils is a hallmark feature of oral submucous fibrosis (OSF). However, the precise characteristics and underlying mechanisms remain unclear, impeding the advancement of potential therapeutic approaches. Here, we observed that collagen I, the main component of the extracellular matrix, first accumulated in the lamina propria and subsequently in the submucosa of OSF specimens as the disease progressed. Using RNA-seq and Immunofluorescence in OSF specimens, we screened the cartilage oligomeric matrix protein (COMP) responsible for the abnormal collagen accumulation. Genetic COMP deficiency reduced arecoline-stimulated collagen I deposition significantly in vivo. In comparison, both COMP and collagen I were upregulated under arecoline stimulation in wild-type mice. Human oral buccal mucosal fibroblasts (hBMFs) also exhibited increased secretion of COMP and collagen I after stimulation in vitro. COMP knockdown in hBMFs downregulates arecoline-stimulated collagen I secretion. We further demonstrated that hBMFs present heterogeneous responses to arecoline stimulation, of which COMP-positive fibroblasts secrete more collagen I. Since COMP is a molecular bridge with Fibril-associated collagens with Interrupted Triple helices (FACIT) in the collagen network, we further screened and identified collagen XIV, a FACIT member, co-localizing with both COMP and collagen I. Collagen XIV expression increased under arecoline stimulation in wild-type mice, whereas it was hardly expressed in the *Comp*^*-/-*^ mice, even with under stimulation. In summary, we found that COMP may mediates abnormal collagen I deposition by functions with collagen XIV during the progression of OSF, suggesting its potential to be targeted in treating OSF.

## Introduction

Oral submucous fibrosis (OSF) is a chronic, insidious oral potentially malignant disorder (OMPD),^1^ with a malignant transformation rate of 7%–13%.^1–3^ The clinical symptoms of OSF, such as pale mucosa, burning sensations, and restricted mouth opening, significantly impair patients’ quality of life.^4,5^ The main pathological characteristic of OSF is abnormal collagen deposition, which impacts both its malignant transformation potential and clinical symptoms.^6–10^ Therefore, understanding the underlying mechanisms of abnormal collagen accumulation during OSF progression is crucial for advancing the development of therapeutic approaches.

In oral mucosa, collagen I fibrils are the most abundant component of all types of collagen.^11,12^ Collagen I fibrils exhibit a hierarchical structure, requiring a multi-stage assembly process.^13^ Procollagen triple helix is secreted into the extracellular space to form collagen, collagen further assemble into microfibrils and finally merge into mature fibrils through longitudinal and axial growth.^13^ The axial growth process of collagen fibrils plays a vital role in forming type I collagen fibrils. During this stage, Fibril-associated collagen with Interrupted Triple helices (FACIT) can integrate into the surface of collagen fibrils that would regulate the axial growth and limit fibril diameter.^14–16^ Collagen XII and collagen XIV, both members of the FACIT family, have been identified as being associated with collagen I fibrils contributing to collagen fibrils architecture.^17–20^ However, whether and how FACIT contributes to the formation of abnormal collagen structures in OSF has not yet been investigated.

COMP is a pentameric multidomain protein, which consists of an N-terminal domain, four type 2 epidermal growth factor (EGF)-like repeats, seven type 3 calcium-binding repeats, and a C-terminal globular domain.^21^ Through its C-terminal domain, COMP can bind to various components of the extracellular matrix (ECM), contributing to organ fibrosis and other pathological conditions.^22^ For example, In the dermis, COMP accumulates in systemic sclerosis skin and keloids, inhibiting COMP can reduce collagen I deposited in vitro.^23,24^ However, the underlying mechanism of which COMP causes collagen I deposition remain unclear. A few in vitro studies reveal that COMP can act as a molecular bridge in the collagen II network by binding to collagen IX, a FACIT that decorates the surface of collagen II fibrils,^25,26^ indicating its role as a molecular bridge in FACIT decoration. However, whether and how COMP functions in the collagen I accumulation during the progression of OSF remains unknown.

In this study, we examined the changes in collagen I accumulation over time and space in OSF specimens during disease progression. We found that collagen I is abnormally accumulated in the lamina propria during the early stage, progressing deeper into the submucosa as the disease progressed. Through RNA sequencing, we identified COMP as a key pathogenic factor in mediating this abnormal accumulation of collagen. Our results suggest that COMP may facilitate abnormal collagen I fibrils deposition by interacting with collagen XIV during the progression of OSF.

## Results

### Spatial-temporal accumulation of collagens in the pathogenesis of OSF

**Fig. 1:**
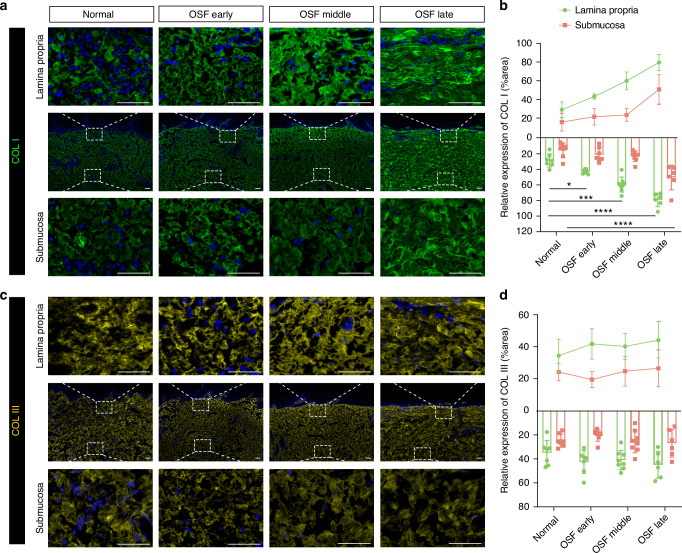
Pathological characteristics of abnormal collagen I and collagen III accumulation during OSF progression. **a** Distribution and expression of collagen I in the lamina propria and submucosa. **b** Immunofluorescence assay of collagen I in the lamina propria and submucosa. **c** Distribution and expression of collagen III in the lamina propria and submucosa. **d** Immunofluorescence assay of collagen III in the lamina propria and submucosa. scale bar = 50 μm, *n* = 28, Data are presented as mean ± SD, *P < 0.05, ***P < 0.001, ****P < 0.000 1

The development of OSF starts with the abnormal accumulation of collagen fibers followed by hyaline degeneration (Fig. S1), indicating that characteristics of collagen accumulation vary throughout the progression of OSF. To depict how collagen deposits over space and time, we examined the micropathological features of major collagen types, collagen I and collagen III, using immunostaining. We observed that the micropathological characteristics of collagen I differed from those of collagen III: In the normal buccal mucosa, collagen I was homogeneously expressed in both the lamina propria and the submucosa. In the early to middle stages, collagen I expression was upregulated in the lamina propria but not in the submucosa. In the late stage of OSF, we observed significant deposits of collagen I in both the lamina propria and submucosa, although the collagen was much denser in the lamina propria (Fig. 1a, b). In contrast, no significant differences were observed in the expression of collagen III in different disease stages (Fig. 1c, d). In summary, collagen I accumulation follows a clear spatial and temporal sequence during the progression of OSF, including early accumulation in the lamina propria and gradual progression to the submucosa.

### Collagen metabolism-related gene COMP may be a key molecule mediating abnormal collagen I accumulation in OSF

**Fig. 2:**
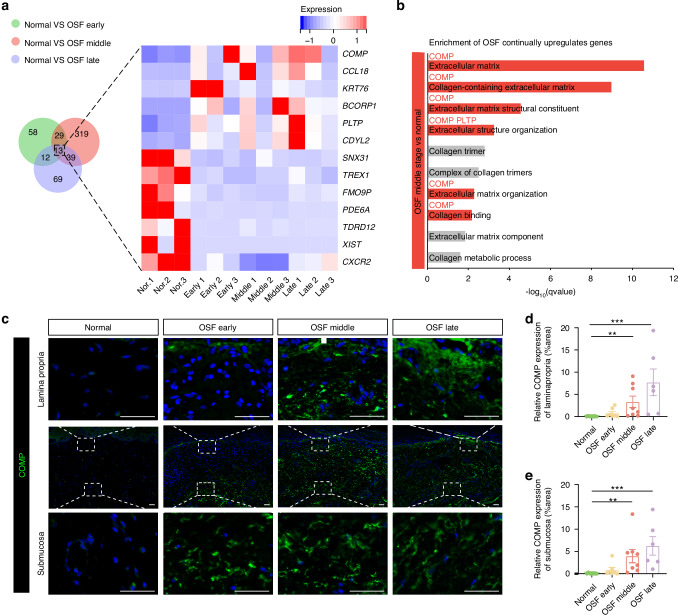
COMP mediates abnormal collagen accumulation during the progression of OSF (**a**) Venn analysis of DEGs between OSF early vs. normal, OSF middle stage vs. normal, and OSF late stage vs. normal. DEGs were analyzed using edgeR software.The criteria for identifying DEGS were a FDR < 0.05 and |log_2_(fold change (FC))| ≥ 1. Heat maps of 13 common DEGs. **b** GO analysis of differential genes in OSF mid-stage compared to the normal group, which contains several GO terms related to extracellular matrix and collagen metabolism. Enrichment of genes with persistent upregulation in OSF. **c** Distribution and expression of COMP in the lamina propria and submucosa, scale bar = 50 μm. **d** Immunofluorescence assay of COMP in the lamina propria (*n* = 28). **e** Immunofluorescence assay of COMP in the submucosa (*n* = 28). Data are presented as mean ± SD, ** *P* < 0.05, *** *P* < 0.001

To investigate the molecular mechanisms that mediate the abnormal accumulation of collagen I, we collected buccal mucosal tissues from patients with early, middle, and late stage OSF and healthy controls for RNA sequencing. Differential mRNA expression profiles in OSF pathogenesis were analyzed (Fig. S2). As we expected, collagen remodeling and metabolism-related genes were consistently upregulated during OSF progression (Supplementary Tables 1, 2). We further intersected differential expressed genes (DEGs) in the early, middle, and late stages of OSF revealing that six genes, including *COMP*, *CCL18*, *KRT76*, *BCORP1*, *PLTP*, and *CDYL2* were consistently upregulated (Fig. 2a). *COMP* enriched in most collagen remodeling-associated GO terms, indicating its crucial role in regulating collagen accumulation during OSF progression (Figs. 2b, S3). In addition, we paid attention to the upregulated DEGs during OSF progression (Figs. S4, S5), and found that COMP may be involved in the ECM-receptor interaction pathway in the middle stage of OSF and the PI3K-Akt signaling pathway in the late stage of OSF (Fig. S4). Therefore, we performed immunostaining of COMP in our OSF-specimen cohort. We showed that the distribution of COMP proteins presents a low level in the lamina propria and submucosa regions of normal samples (Fig. 2c–e). However, the expression of COMP proteins was significantly upregulated in the lamina propria and submucosa during OSF progression (Fig. 2c–e), indicating critical functional involvement of abnormal activation of COMP during OSF progression. Thus, we next focus on the role of COMP in influencing collagen I accumulation during OSF progression.

### COMP deficiency reduces arecoline-stimulated accumulation of collagen I

**Fig. 3:**
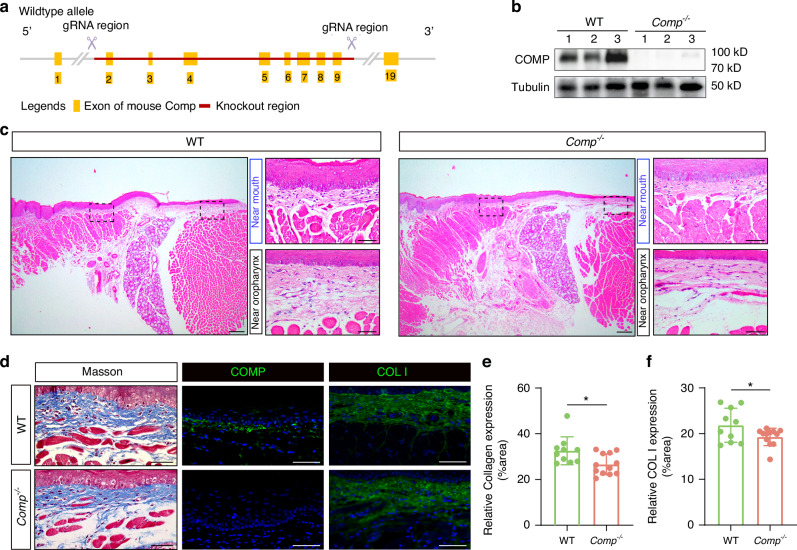
Construction and phenotypic observation of *Comp*^*-/-*^ mice (**a**) Generation of *Comp*^*-/-*^ mouse model (C57BL/6N) using CRISPR/Cas-mediated genome engineering, an overview of the targeting strategy. **b** Expression of COMP protein in its positive reference mouse cartilage. (**c**) H&E staining in the buccal mucosa of WT and *Comp*^*-/-*^ mice (left, scale bar = 200 μm; right, scale bar = 50 μm). **d** Masson’s trichrome stain and immunofluorescence stain for collagen I and COMP in buccal mucosa of WT and *Comp*^*-/-*^ mice (scale bar = 50 μm). **e** Masson’s trichrome stain assay of WT and *Comp*^*-/-*^ mice. **f** Immunofluorescence assay of WT and *Comp*^*-/-*^ mice. *n* = 22, Data are presented as mean ± SD, *P < 0.05

To validate whether COMP mediates abnormal collagen accumulation, we constructed a *Comp*^*-/-*^ mouse model (C57BL/6 N) using CRISPR/Cas (Fig. 3a). No COMP expression was observed in *Comp*^*-/-*^ mice, indicating effective COMP disruption in the *Comp*^*-/-*^ mouse model (Fig. 3b, d). The *Comp*^*-/-*^ mice exhibited normal growth and development (Fig. S6), and no apparent abnormalities were observed in the histological structure of their buccal mucosa (Fig. 3c). However, Masson’s trichrome staining and collagen I immunofluorescence showed that *Comp*^*-/-*^ mice had slightly less total collagen and collagen I than WT mice (Fig. 3d–f).

**Fig. 4:**
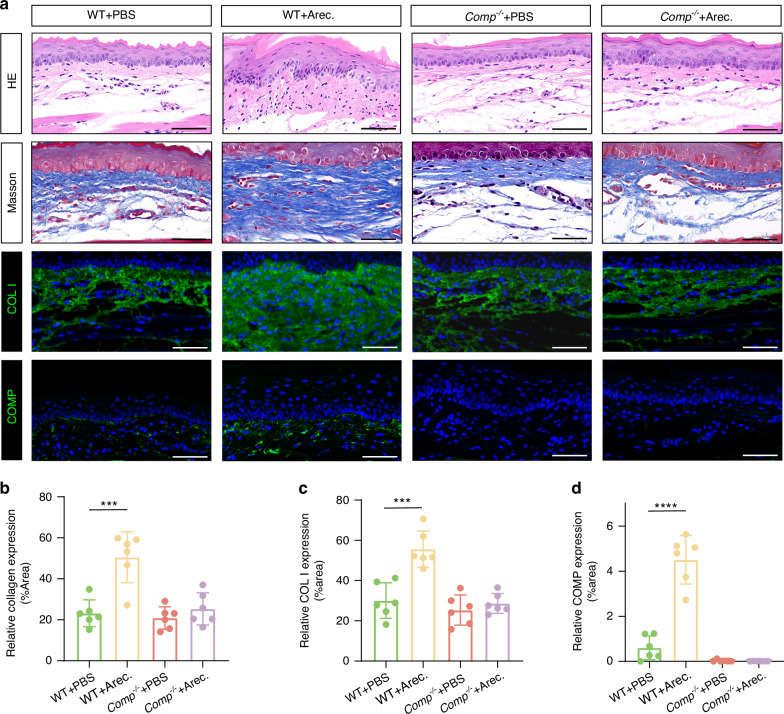
The absence of COMP may reduce the degree of collagen I accumulation stimulated by arecoline. **a** Histological assessments using H&E, Masson, and immunofluorescence staining for collagen I and COMP in the buccal tissues of mice treated with PBS and arecoline, scale bar = 50 μm. **b** Masson’s trichrome stain assay of the buccal tissues of mice treated with PBS, and arecoline. **c**, **d** Immunofluorescence assay of the buccal tissues of mice treated with PBS, and arecoline. *n* = 24, Data are presented as mean ± SD, ***P < 0.01. ****P < 0.001

We next investigated the impact of COMP disruption during OSF progression. Chewing areca nuts is a significant risk factor for OSF.^27,28^ Previous studies have revealed that injecting arecoline, a key component in areca nuts, into the buccal mucosa of mice develops OSF.^29–32^ Therefore, to further validate the role of COMP in the abnormal deposition of collagen I, we injected arecoline (20 mg/kg) locally into the buccal mucosa of WT mice and *Comp*^*-/-*^ mice daily for 12 weeks, and injected an equivalent volume of phosphate buffered saline (PBS), the solvent used with arecoline, for the control group. Collagen deposition was evaluated using hematoxylin and eosin (H&E) and Masson’s trichrome staining and collagen I immunofluorescence. The results showed that abnormal collagen deposition was significantly induced in the buccal mucosa of WT mice with arecoline injection compared to that in the control group, whereas abnormal collagen deposition was not observed in *Comp*^*-/-*^ mice injected with either arecoline or PBS (Fig. 4a–c). COMP expression was upregulated in the WT mice injected with arecoline compared to those injected with PBS(Fig. 4a, d). These data uncover that COMP may play a crucial role in the deposition of collagen I.

### Arecoline stimulates collagen I deposition in fibroblasts via COMP

**Fig. 5:**
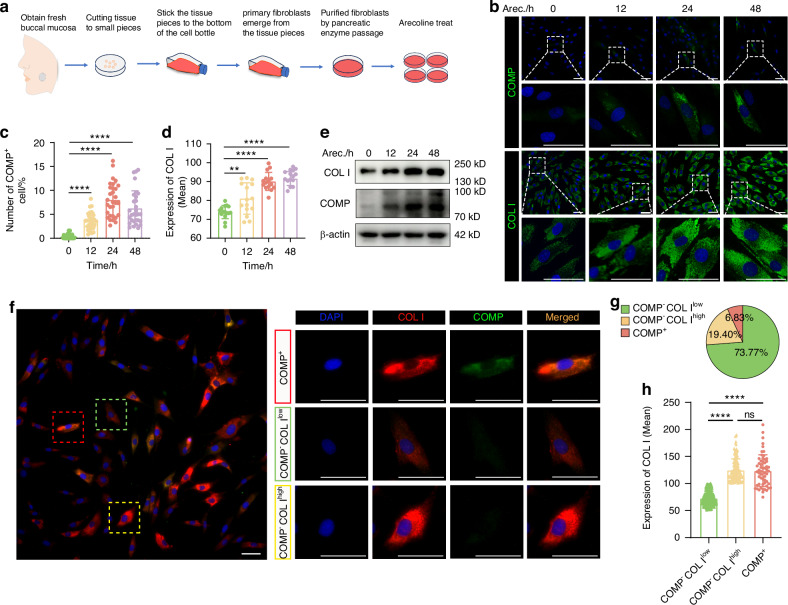
COMP promotes abnormal collagen accumulation in hBMFs. **a** Schematic diagram of hBMFs cultured in the buccal mucosa. **b** Immunofluorescence staining depicts COMP and collagen I expression in hBMFs stimulated by arecoline. **c** Immunofluorescence assay quantifying the number of COMP-positive cells (*n* = 120). **d** Immunofluorescence assay quantifying the expression of collagen I (*n* = 60). **e** Western blot analysis of collagen I, and COMP in hBMFs treated with arecoline. **f** Immunofluorescence co-staining of COMP and collagen I following arecoline stimulation. **g** Percentage distribution of three cell types. **h** Immunofluorescence assay of the expression of collagen I in three cell types; *n* = 835. Data are presented as mean ± SD, ns not significant, **P < 0.01, ****P < 0.000 1

We next focused on how abnormal COMP expression is activated during OSF progression. As human buccal mucosal fibroblasts (hBMFs) are the main cell type producing collagen in OSF,^33–35^ we estimate that hBMFs may be the source of abnormal COMP expression. To investigate whether hBMFs can be abnormally induced to upregulate COMP thus causing collagen I deposition, we isolated normal hBMFs and treated them with arecoline (Figs. 5a, S7). Under normal conditions, very few fibroblasts expressed COMP (Fig. 5b, c). Upon arecoline stimulation, the expression levels of both COMP and collagen I were upregulated time-dependent (Figs. 5b, d, e, S8). In addition, the number of COMP-positive fibroblasts gradually increased under stimulation and was the highest at 24 h post arecoline stimulation (Fig. 5b, c). Interestingly, Immunofluorescence co-localization revealed that hBMFs present heterogeneous response to arecoline treatment. COMP-positive fibroblasts exhibit elevated COMP expression and secrete higher levels of collagen I. (Fig. 5f). Due to the heterogeneous expression level of collagen I within COMP-negative fibroblasts, we classified them into collagen I ^high^ and collagen I ^low^ fibroblasts using the lower quartile of collagen I expression intensity as the threshold. Quantitative analysis showed that most COMP-negative fibroblasts had low collagen I levels (Fig. 5g, h). These results indicate that high COMP expression in fibroblasts correlates with high collagen I production, suggesting the intracellular function of COMP in collagen I secretion.

**Fig. 6:**
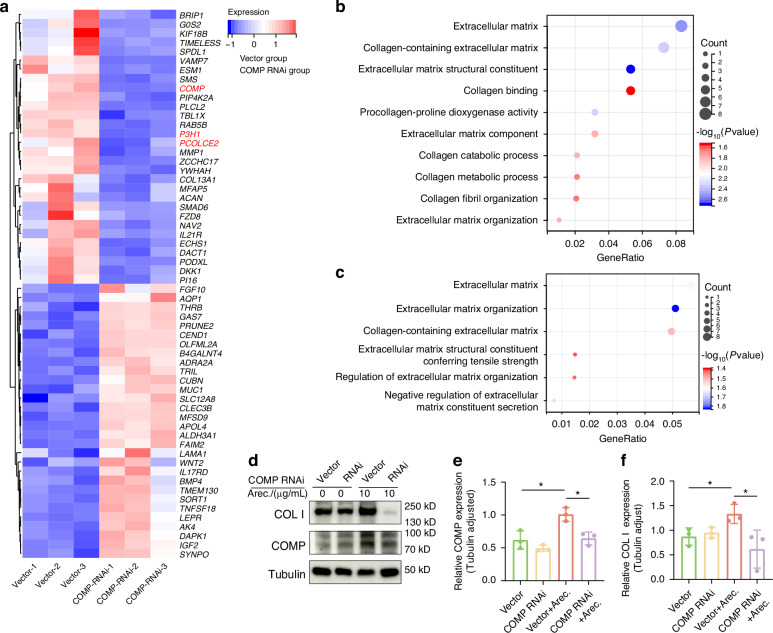
Fibroblasts that knocked down COMP had a reduced ability to synthesize collagen. **a** Heatmap of the top 30 upregulated DEGs and downregulated DEGs between shCOMP hBMFs and vector group. The gene in red is closely related to collagen assembly. **b**, **c** GO enrichment analysis of downregulated DEGs (**b**) and upregrauted DEGs (**c**) in shCOMP hBMFs compared to the vector group, which contains several GO terms related to extracellular matrix and collagen metabolism. **d**–**f** Western blot analysis of collagen I and COMP in shCOMP hBMFs treated with arecoline. Data are presented as mean ± SD, *P < 0.05

To explore the effect of COMP on fibroblast function, we knocked down COMP in hBMFs and performed RNA sequencing, we observed a significant reduction in prolyl 3-hydroxylase 1(P3H1) and procollagen C-endopeptidase enhancer 2(PCOLCE2) expression. Both of them are essential for collagen assembly (Fig. 6a). Additionally, GO enrichment analysis indicated abnormal functions associated with collagen remodeling in the extracellular matrix following knock down COMP (Fig. 6b, c). To validate whether activation of COMP expression upregulates collagen I production, we stimulated the hBMFs with arecoline and found that collagen I synthesis was greatly reduced following the silencing of COMP, particularly following stimulation by arecoline. Meanwhile, arecoline promoted the secretion of COMP and collagen I in control fibroblasts (Fig. 6d–f).

### Collagen XIV, not collagen XII, modifies collagen I during collagen deposition in OSF

**Fig. 7:**
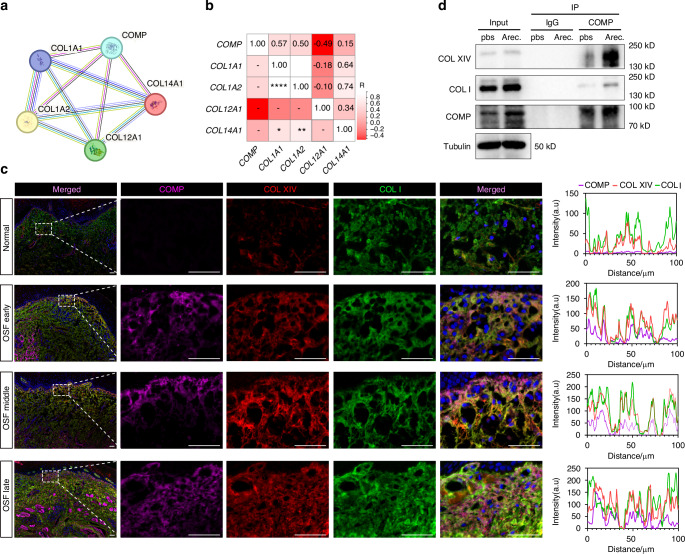
Collagen XIV, but not collagen XII, modifies collagen I to promote abnormal collagen accumulation during OSF pathogenesis. **a** A protein interaction network derived from the string database. **b** Correlation heatmap of mRNA levels from OSF clinical samples. **c** Immunofluorescence co-localization of COMP, collagen XIV and collagen I in human buccal mucosal tissues, scale bar = 50 μm. **d** co-immunoprecipitation detected the interaction between COMP and collagen I and collagen XIV

Previous studies indicate the role of COMP as a extracellular molecular bridge in the collagen II network by modifying the surface of collagen IX,^25,26^ indicating it may have similar function in collagen I deposition. STRING protein interactions revealed that COMP may interact with COL12A1 and COL14A1, both of which belong to the FACIT family (Fig. 7a). The correlation heatmap showed that, compared to COL12A1, COMP was more strongly correlated with COL14A1 (Fig. 7b). Immunofluorescence showed that the expression level of collagen XIV was elevated during OSF development, and the collagen XIV protein largely co-localized with collagen I (Fig. S9, S10). Notably, collagen XIV expression in the lamina propria was more significantly upregulated than that in the submucosa, similar to the patterns of collagen I and COMP (Fig. 1a, b, Fig. 2c–e, Fig. S9, S10). In contrast, the expression level of collagen XII was much lower than that of collagen XIV, and co-localization with collagen I was rarely observed (Fig. S9, S11). These results indicate that collagen XIV, not collagen XII, modifies collagen I to promote abnormal collagen deposition during OSF pathogenesis.

### COMP mediates the arecoline-induced secretion of collagen I and collagen XIV

To verify whether COMP is involved in the formation of collagen XIV and collagen I collagen structures that facilitate the progression of OSF, we first found their local co-localization through immunofluorescence techniques(Fig. 7c). The interaction between COMP, collagen XIV and collagen I was subsequently confirmed via co-immunoprecipitation experiment (Fig. 7d).

**Fig. 8:**
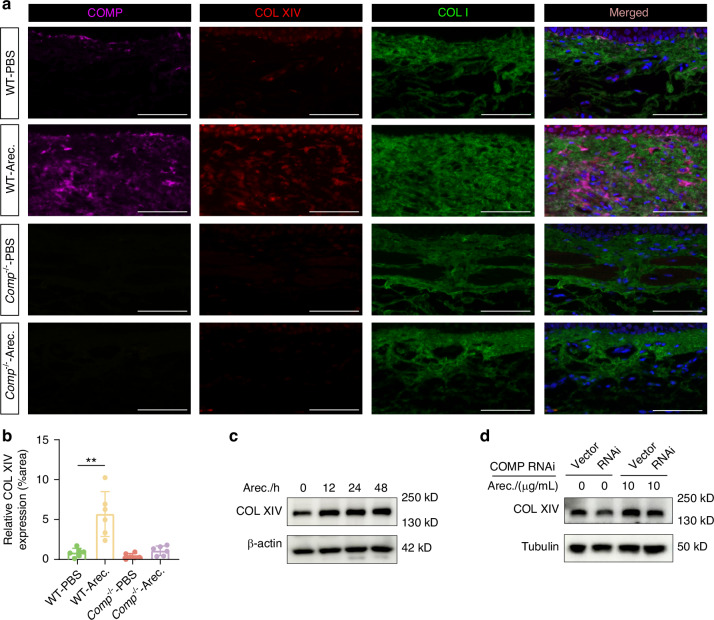
Arecoline promotes the binding of COMP to COL XIV, which modifies collagen I. **a** Immunofluorescence co-localization of COMP, collagen XIV and collagen I in mouse buccal mucosal tissues. scale bar = 50 μm. **b** Immunofluorescence analysis of collagen XIV expression in mouse buccal tissues. *n* = 24. **c** Western blot analysis of collagen XIV in hBMFs treated with arecoline. **d** Western blot analysis of collagen XIV in shCOMP hBMFs treated with arecoline. Data are presented as mean ± SD, **P < 0.01

To determine whether collagen XIV can be regulated by arecoline stimulation, we analyzed the expression levels of collagen XIV in the buccal mucosa of WT mice with or without arecoline treatment. Collagen XIV expression was upregulated following arecoline stimulation (Fig. 8a, b). Meanwhile, we observed co-localization of COMP, collagen XIV, and collagen I in the arecoline-stimulated buccal mucosa of WT mice (Fig. 8a). At the cellular level, collagen XIV expression was upregulated in arecoline-stimulated fibroblasts in a time-dependent manner in vitro (Figs. 8c, S12a).

We then studied whether arecoline- stimulated secretion of collagen I and collagen XIV were mediated by COMP. We found that collagen XIV expression in the buccal mucosa of *Comp*^*-/-*^ mice was not elevated following arecoline treatment (Fig. 8a, b). At the cellular level, following COMP knockdown, the expression of collagen XIV was not significantly elevated, even in the presence of arecoline stimulation (Figs. 8d, S12b).

## Discussion

In this study, we observed a sequential accumulation of collagen I in the lamina propria at an early stage and later in the submucosa as the disease progressed. Through RNA sequencing, we identified COMP as a crucial causative factor in the abnormal deposition of collagen. By constructing *Comp*^*-/-*^ mice, we demonstrated that deleting COMP reduced the extent of arecoline-stimulated collagen I deposition. Given that COMP usually binds to FACITs and acts as a molecular bridge in the collagen network, we found that collagen XIV, a member of FACIT, was co-localized with COMP and collagen I. Functional experiments showed that collagen XIV expression was upregulated in response to arecoline stimulation in WT mice, whereas it was hardly expressed in *Comp*^*-/-*^ mice, even under stimulation. These findings suggest that COMP may mediate abnormal collagen I deposition by interacting with Collagen XIV during the progression of OSF.

Abnormal collagen accumulation is associated with an abnormal collagen structure. The skin of patients with scleroderma exhibits a large number of thin collagen fibrils, either in bundles alone or intermingled with large-diameter fibrils.^36^ During the early stages of OSF, a transition occurs from uniformly sized collagen fibril bundles to the presence of immature, fine fibrils within the interfibrous matrix. As the disease progresses, the diameter of the fiber bundles varies, leading to a dense array of fibers.^37,38^ The fibrous network structure of the ECM in the oral mucosa is composed of a variety of macromolecules, primarily collagen I and III. While collagen I levels gradually increase with the progression of OSF, previous research has not clarified the spatial and temporal dynamics of collagen I accurately.^7,9^ Our study, however, found for the first time that collagen I abnormally accumulates in the lamina propria in the early stage of OSF and subsequently penetrates deeper into the submucosa as the disease progresses.

COMP is an ECM glycoprotein that is involved in fibrosis, skeletal diseases, cancer, and cardiovascular diseases.^21,22^ In fibrotic diseases, COMP expression levels often correlate with disease progression across different disorders, including scleroderma,^23,39–41^ skin keloid,^24,40^ liver fibrosis^42,43^ and idiopathic pulmonary fibrosis.^44^ In skin keloids, COMP is expressed at higher levels in larger lesions but at lower levels in small scars.^24^ Similarly, in idiopathic pulmonary fibrosis, COMP is expressed at higher levels in areas of dense fibrosis.^44^ The upregulation of COMP stimulates the accumulation of collagen and other ECM proteins, exacerbating fibrosis severity.^23,39,45,46^ In this study, we found that COMP expression was upregulated in both the lamina propria and submucosa of OSF samples. Further, we found that COMP mediates abnormal collagen I deposition.

The observation of the pathological changes in the abnormal structure of the collagen fibrils network prompted us to investigate the process of collagen fibrils formation. Collagen categories include classical fibrillar collagen, FACITs, and membrane-associated collagens with interrupted triple helices (MACITs), etc. ^47^ Fibrillar collagen is the main component of the ECM in the connective tissues, and collagen I is the most abundant fibril of the oral mucosa.^48^ The structure of FACITs is characterized by “collagenous domains” interrupted by short non-helical domains, which play a role in modifying the surfaces of classical fibrillar collagen fibrils. For example, collagen IX, a member of FACIT, is associated with collagen II fibrils, while collagen XII and collagen XIV modifies the surface of collagen I fibrils.^18,49^ FACIT also interacts with other ECM molecules, such as COMP.^20,50–52^ COMP acts as a molecular bridge in the collagen II network by binding to collagen IX in cartilage.^21,22,53^ In addition, COMP binds to collagen XII and XIV, which associate with collagen I fibrils in the ECM of the skin.^20^ In our study, we found that collagen XIV, a member of the FACIT family, correlated with collagen I and co-localized with collagen I and COMP in the oral mucosa. These results suggest that COMP mediates abnormal collagen I deposition by serving as a molecular bridge interacting with collagen XIV.

COMP may play different roles during the progress of OSF. COMP’s interaction with collagen I and XIV was significantly increased in OSF tissues, helping to explain the observed collagen deposition. Interestingly, by visualizing the co-localization of COMP and collagen I in arecoline-stimulated fibroblasts, we found that COMP-positive fibroblasts had relatively high collagen I expression, which indicates COMP promotes collagen synthesis intracellularly. These results indicate the dual intracellular and extracellular roles of COMP, although its specific mechanism remains to be further studied. As OSF advances to the late stage, the cellular population diminishes significantly, and COMP may primarily function as an organizer of collagen fibers.

TGF-beta signaling is critical for oral submucosal fibrosis. TGF-beta was associated with fibrotic disease by induces COMP expression. In skin fibrosis and lung fibrosis, COMP regulate the TGF-beta signaling pathway, and both COMP and TGF-beta form a positive feedback loop.^44,54^ Furthermore, COMP promotes the progression of fibrosis by interacting with CD36 and activating the MEK/ERK and PI3K/AKT signaling pathways.^46,55–57^ COMP has intracellular functions that facilitate collagen secretion of dermal fibroblasts.^45^ In COMP-deficient fibroblasts, ER stress is caused by collagen retention in the ER.^45^ In this study, although we revealed the possible intracellular and extracellular roles of COMP, the detailed regulation mechanisms of COMP mediated collagen synthesis or deposition needs further studies.

This study has some limitations. First, the quantification of COMP expression level is needed in a larger cohort of OSF patients. Second, given that COMP expression was only in part of hBMFs, future studies based on single cell sequencing from controls and OSF patients at different stages will help us to identified the characterized fibroblast subset expressing COMP. Third, this study did not provide detailed quantitative data on collagen I expression levels across different OSF stages in vivo in mice, which would help assess the progression’s severity and translate the results to humans. Last, this study did not investigate additional potential factors like TGF-beta or compensatory mechanisms that may influence collagen deposition other than COMP.

This study focused on the abnormal assembly of collagen structures during OSF progression and revealed that COMP functions as an important mediator of this process. Given that COMP expression can be upregulated by arecoline, it suggests that COMP-mediated collagen deposition may be a key pathological mechanism underlying OSF. Therefore, COMP may function as a significant biomarker of OSF. Furthermore, targeting COMP is a promising treatment for OSF, and future studies should focus on investigating potential small molecules that could inhibit the function of COMP.

## Materials and methods

### Patient tissue samples

The study protocol was conducted in accordance with the principles of the Declaration of Helsinki and approved by the Research Ethics Board of the Scientific and Ethical Committee of Xiangya Hospital (Approval numbers 2019030562 and 2024020145). Mucosal tissues were obtained after written informed consent was obtained from the patients. Normal tissues were obtained from patients with low impacted tooth extraction and buccal mucosa urethral reconstruction, and OSF samples were obtained from the biopsy samples of the patients during their first visit, the final diagnosis was made by professional pathologists. The pathological manifestations of OSF at different stages are as follows. In the early stage of OSF, a large number of inflammatory cells were infiltrated and collagen fiber edema was observed, but no significant fibrotic changes were observed. In the middle stage, the connective tissues show hyalinization of collagen, with mild edema and inflammatory cell infiltration. In the late stage, collagen fibers are severely hyalinized, the number of fibroblasts is significantly reduced, inflammatory cell infiltration is almost invisible, and blood vessels are narrowed or blocked. The clinical characteristics of the patients are shown in Supplementary Tables 3 and 4.

### Pathological observation

Tissues were fixed in 4% paraformaldehyde overnight, dehydrated using an ethanol gradient, embedded in paraffin, and sectioned into 4-μm sections. H&E staining was performed, and the sections were embedded in neutral resin and photographed using a Leica DM750 light microscope (Leica Microsystems, Wetzlar, Germany).

Total collagen accumulation was evaluated using a Modified Masson’s trichrome staining kit (G1346, Solarbio, Beijing, CHN). Images were captured using a Leica DM750 light microscope (Leica Microsystems, Wetzlar, Germany). Collagen fibers are shown in blue, cytoplasm and muscle tissue are shown in red, and nuclei are shown in blue-brown. The area of the collagen volume fraction was determined using ImageJ software.

### Immunofluorescence assay

Paraffin-embedded samples were sectioned into 4-µm-thick slices, dried at 60 °C for 2 h, dewaxed in xylene, and hydrated using the gradient ethanol method. The antigen was microwave-repaired and blocked with goat serum for 30 min. Primary antibodies were added and kept overnight at 4 °C, then the tissue was incubated with the appropriate secondary antibody at room temperature for 1 h, and the tablet was sealed with DAPI-containing anti-quenching sealing tablets. Images were captured using a Leica DM6B Thunder fluorescence microscope (Leica Microsystems, Wetzlar, Germany) and analyzed using ImageJ software. The primary antibodies used were as follows: collagen I (CST, Danvers, MA, USA; #72026; 1:200), collagen III (Proteintech, Wuhan, China; 22734-1-AP, 1:200), COMP (Abcam, Cambridge, MA, USA; ab300055, 1:1 000), collagen XIV (Invitrogen, Carlsbad, CA, USA; PA5-49916, 1:150), and collagen XII (Santa Cruz Biotechnology, Santa Cruz, CA,USA; sc-166020, 1:200).

A tyramide signal amplification (TSA) kit (AFIHC034; Ai Fang biological, Changsha, China) was used for multiple immunofluorescence tests: (1) Each slide was dewaxed using xylene; (2) Antigen repair was the same as mentioned above; (3) A 3% hydrogen peroxide solution was used to block endogenous peroxidases; (4) Goat serum was used as a non-specific target; (5) The primary antibody was incubated at 4 °C overnight; (6) The poly-horseradish peroxidase (HRP) secondary antibody was incubated at room temperature for 50 min; (7) The TSA fluorescent dye reaction solution was administered under a fluorescence microscope; (8) Antibodies were eluted; (9) Steps 3–8 were repeated (replaced by the second TSA fluorescent dye label); 10) Steps 3–7 were repeated (replaced by the third TSA fluorescent dye label); (11) Slides were mounted with DAPI-containing Prolong Gold antifade reagent (P36935, Invitrogen); (12) Images were captured using a Leica DM6B Thunder fluorescence microscope (Leica Microsystems, Wetzlar, Germany).

### Animal studies

*Comp*^*-/-*^ mice were generated using CRISPR/Cas9-mediated genome editing. *Comp* (NCBI reference sequence: NM_016685; Ensembl: ENSMUSG00000031849) is located on mouse chromosome 8. Nineteen exons have been identified in mouse chromosome 8, with an ATG start codon in exon 1 and a TAG stop codon in exon 19 (Transcript: ENSMUST0000000003659). Exons 2–9 were selected as target sites. Heterozygous mutant mice were mated with homozygous mutant mice to generate heterozygous mutant mice, which were intercrossed to produce homozygous mutant mice. The mice were identified by PCR, and the genotyping primers used are listed in Supplementary Table 5. Western blotting of the positive tissue (mouse cartilage) was used to verify the COMP knockout.

Animal experiments were approved by the Animal Ethics Committee of the Department of Laboratory Animals, Central South University, and. housed at the Central South University Department of Laboratory Animal Science, Changsha, China. At 7 weeks of age, the mice were randomly allocated into the WT + PBS, WT+ arecoline, *Comp*^*-/-*^+PBS, and *Comp*^*-/-*^+arecoline groups.

Arecoline was diluted to 4 mg/mL in PBS immediately before injection. The WT+ arecoline and *Comp*^*-/-*^+arecoline groups were treated with a regional local injection of arecoline (20 mg/kg, daily) on the left cheek using a 30G insulin syringe (KDL, Wenzhou, China). An equal volume of PBS was administered to the control group. After 12 weeks of treatment, the mice were euthanized and buccal tissues were collected.

### Cell Culture

Primary fibroblasts were isolated from normal human buccal tissue using a nonenzymatic procedure. Tissue samples were inserted into 15-mL polypropylene tubes containing 5 mL Dulbecco’s modified Eagle’s medium (DMEM) (Gibco, Carlsbad, CA, USA) with 3% penicillin-streptomycin. The tissues were processed as quickly as possible after surgery. Tissue samples were rinsed three times in a cell culture dish with PBS containing 3% penicillin-streptomycin. The tissue samples were finely chopped into 1 mm 2 pieces using sterile scissors. A T25 cell culture flask (Nest, China) was washed with DMEM, the medium was removed, and the tissue pieces were gently pressed into the T25 flask. The tissue pieces were dried for 4 h in a humidified 5% CO_2_ incubator at 37 °C. Fibroblasts between passages 3 and 8 were cultured in DMEM containing 10% fetal bovine serum and 1% penicillin-streptomycin in a humidified 5% CO_2_ incubator at 37 °C.

### Stable cell line generation

LV-COMP-RNAi recombinant lentivirus and GV112 vector lentivirus were purchased from GeneChem (Shanghai, China). The lentivirus design is illustrated in Supplementary Table 6. Fibroblasts were infected with lentivirus (MOI = 10) according to the manufacturer’s protocol. Selective culture medium containing puromycin (2 µg/mL) was used to select the stable expression cells. COMP expression was detected using western blotting.

### Cell Immunocytochemistry

The fibroblasts were cultured on coverslips, fixed with 4% paraformaldehyde and permeabilized with 0.1% Triton X-100 in PBS for 20 min, blocked in 3% bovine serum albumin for 1 h, and incubated with primary antibody overnight at 4 °C. The primary antibodies used were against collagen I (CST, Danvers, MA, USA; #72026; 1:200) and COMP (Abcam, Cambridge, MA, USA; ab300055, 1:1 000). The fibroblasts were then washed with PBS, and incubated with Alexa Fluor 488 or 594 secondary antibodies (Abcam, Cambridge, MA, USA; ab150080, ab150077,1:600) for 1 h at room temperature. Then, the coverslips were washed and mounted with histology mounting medium with DAPI (sigma-aldrich, Louis Missouri, FLORIDA, USA; F6057). Images were acquired using a Leica DM6B fluorescence microscope (Leica Microsystems, Wetzlar, Germany).

### Co-immunoprecipitation

hBMFs was treated with arecoline (10 μg/mL) or PBS and exocytosis inhibitor EXO1 for 24 h, Cultured cells were lysed on ice in NP40 buffer containing protease inhibitors. The whole cell lysate was transferred to 1.5 mL centrifuge tube and centrifuged at 12 000 r/min at 4 °C for 15 min. The liquid supernatant was collected and the protein concentration was measured by BCA kit (Thermo Fisher Scientific, MA, USA; 23225). Five percent of the protein sample was taken as the input group. Protein sample (2 mg) was taken from PBS and AREC group for follow-up experiment. The sample was divided into two parts. One part was added with magnetic beads coupled with target antibodies, and the other part was added with magnetic beads coupled with IgG antibodies as control group. After being rotated at 4 °C overnight, the samples were washed with TBST with magnetic beads for three times. Finally, 50 μL elution buffer was added and treated for 5 min at 95 °C.

### Western blot

Cultured cells and mouse cartilaginous tissues were homogenized and lysed on ice in lysis buffer containing protease inhibitors. Protein samples were isolated using a 7.5% sodium dodecyl-sulfate polyacrylamide gel electrophoresis (SDS–PAGE) gel and transferred to polyvinylidene difluoride membranes. The samples were then blocked with 5% skim milk powder for 1 h at room temperature and incubated with collagen I (Proteintech, Wuhan, China; 22734-1-AP, 1:2 000), COMP (Abcam, Cambridge, MA, USA; ab300055, 1:1 000), collagen XIV (Invitrogen, Carlsbad, CA, USA; PA5-49916, 1:1 000), β-actin (Zenbio, Chengdu, China; 250136, 1:5 000) antibodies overnight at 4 °C. HRP-conjugated secondary antibodies were added and incubated at room temperature for 1 h.

### RNA extraction, library preparation, and sequencing

RNA sequencing was performed on human buccal mucosa tissues, including the healthy control group, OSF early-stage group, OSF middle-stage group, and OSF late-stage group; each group contained three independent samples. Total RNA was extracted using the mir Vana miRNA isolation kit (Cat#AM1561, Ambion, Waltham, Ma,USA). Total RNA was analyzed using a NanoDrop ND-2000 spectrophotometer (Thermo Fisher Scientific, Waltham, MA, US) and an Agilent Bioanalyzer 2100 (Agilent Technologies, Santa Clara, CA, US). The cDNA libraries were sequenced using an Illumina sequencing platform (Genedenovo Biotechnology Co. Ltd., Guangzhou, China). Raw reads obtained by sequencing were filtered using Seqtk, Genome mapping of the preprocessed reads was performed using the spliced mapping algorithm in Hisat2 version 2.0.4. Subsequently. DEGs were analyzed using edgeR software. The criteria for identifying DEGS were a false discovery rate (FDR) < 0.05 (the p-value adjusted by the Benjamini–Hochberg method) and |log_2_(fold change (FC))| ≥ 1. Part of the data was analyzed using the bioinformatics analysis website sangerbox.^58^

### Bioinformatics analysis

Statistical analyses were performed using GraphPad Prism 8.0 software. Unpaired two-tailed t-tests were used to determine the significance of the differences between two groups. Analysis of variance (ANOVA) with Tukey’s Honestly Significant Difference (Tukey’s HSD) tests were used to compare more than two groups. The Pearson correlation coefficient was used for correlation analysis. All data are presented as mean ± standard deviation (SD). *P* < 0.05 indicated statistical significance (*P < 0.05, **P < 0.01, ***P < 0.001, ****P < 0.000 1).

## Supplementary information

Supplementary Tables

supplementary figures

Supplementary Table 1-2 GO
