# Supplementary material for: Abnormal collagen deposition mediated by cartilage oligomeric matrix protein in the pathogenesis of oral submucous fibrosis
Source: Int J Oral Sci. 2025 Mar 27;17:25. doi: 10.1038/s41368-025-00355-x (PMC11950347; doi:10.1038/s41368-025-00355-x)
Supplement: Supplementary file 1 — Supplementary Tables [file 41368_2025_355_MOESM1_ESM.docx]

**Supplementary Tables**

Supplementary Table 3. Clinicopathological characteristics of

28 clinical samples for immunofluorescence.

| Characteristics | Normal  (n=7)  number | OSF early stage  (n=7)  number | OSF middle stage  (n=8)  number | OSF late stage  (n=6)  number |
| --- | --- | --- | --- | --- |
| Gender |  |  |  |  |
| Male | 2 | 6 | 8 | 6 |
| Female | 5 | 1 | 0 | 0 |
| Age |  |  |  |  |
| 20-40 | 4 | 2 | 1 | 4 |
| 40-60 | 2 | 4 | 8 | 2 |
| 60-70 | 1 | 1 | 0 | 0 |
| Betel quid chewing |  |  |  |  |
| Never | 7 | 0 | 0 | 0 |
| Ever | 0 | 7 | 8 | 6 |

Supplementary Table 4. Clinicopathological characteristics of

12 clinical samples for RNA sequencing.

| **Characteristics** | **Normal**  (n=7)  number | **OSF early stage**  (n=7)  number | **OSF middle stage**  (n=8)  number | **OSF late stag**e  (n=6)  number |
| --- | --- | --- | --- | --- |
| Gender |  |  |  |  |
| Male | 1 | 3 | 3 | 3 |
| Female | 2 | 0 | 0 | 0 |
| Age |  |  |  |  |
| 20-40 | 1 | 2 | 2 | 2 |
| 40-60 | 2 | 1 | 1 | 1 |
| 60-70 | 0 | 0 | 0 | 0 |
| Betel quid chewing |  |  |  |  |
| Never | 3 | 0 | 0 | 0 |
| Ever | 0 | 3 | 3 | 3 |

Supplementary Table 5. Primers used for PCR

| **primer** | **Sequence 5’-3’** |
| --- | --- |
| Forward primer (F1) | CTGCCTGCGTTCTAGTGCTC |
| Forward primer (F2) | GGACCAGACCTTCTTGGAGATG |
| Reverse primer (R1) | TATCACTGTCCGAGTTACGCTG |

Supplementary Table 6. Easy-siRNA design

| **NO.** | **Accession** | **Target Seq** | **CDS** | **GC%** |
| --- | --- | --- | --- | --- |
| COMP-RNAi | NM_000095 | GATGCTTGTGACAGCGATCAA | 37..2310 | 47.37% |
| Description | Homo sapiens cartilage oligomeric matrix protein (COMP), mRNA | | | |
