# Supplementary material for: Abnormal collagen deposition mediated by cartilage oligomeric matrix protein in the pathogenesis of oral submucous fibrosis
Source: Int J Oral Sci. 2025 Mar 27;17:25. doi: 10.1038/s41368-025-00355-x (PMC11950347; doi:10.1038/s41368-025-00355-x)
Supplement: Supplementary file 2 — supplementary figures [file 41368_2025_355_MOESM2_ESM.pdf]

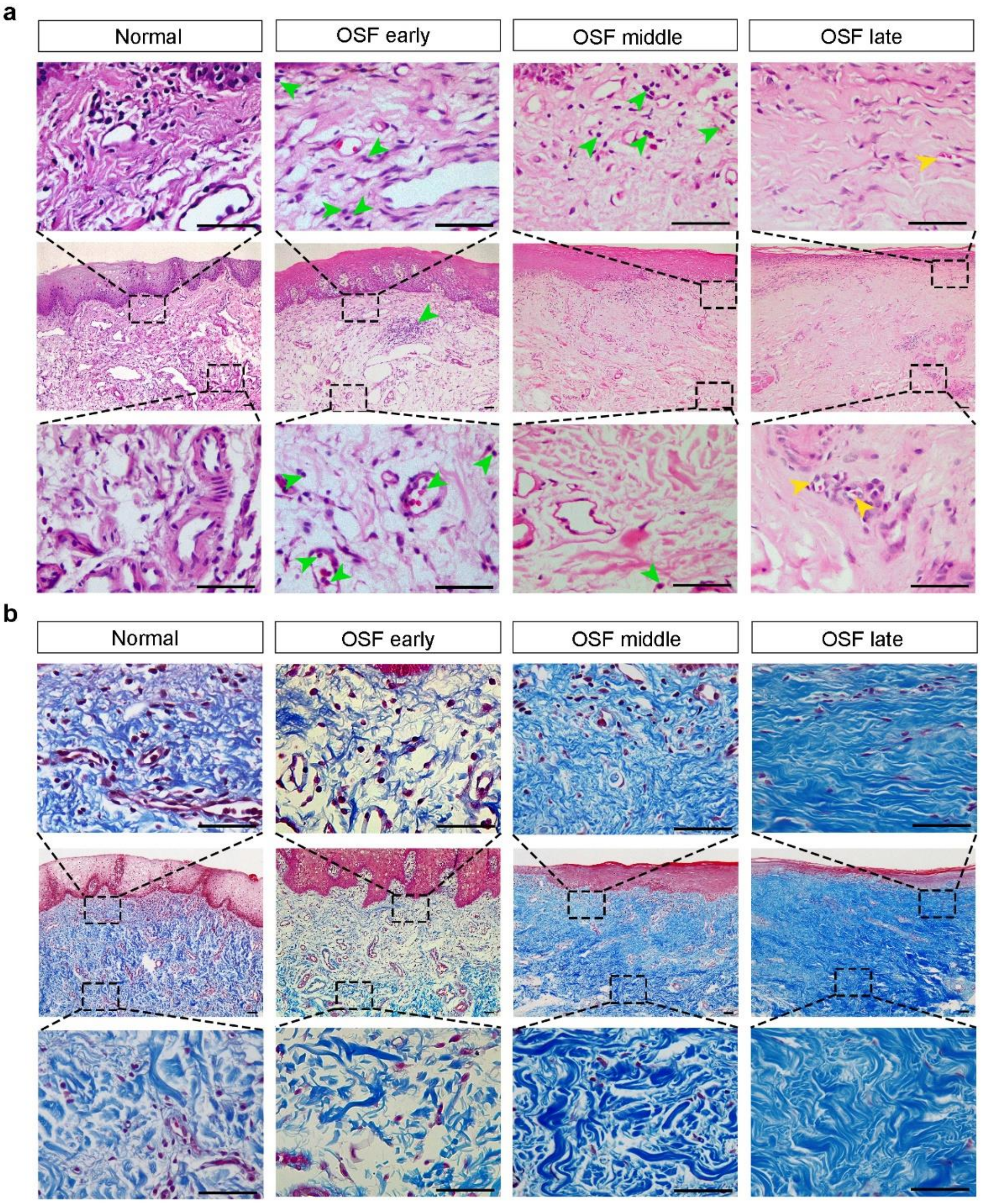


**Figure S1** Pathological characteristics during OSF progression. **a** H&E staining in the lamina propria and submucosa. In the early stage of OSF, a large number of inflammatory cells were infiltrated and collagen fiber edema was observed, but no significant fibrotic changes were observed. In the middle stage, the connective tissues show hyalinization of collagen, with mild edema and inflammatory cell infiltration. In the late stage, collagen fibers are severely hyalinized, the number of fibroblasts is significantly reduced, inflammatory cell infiltration is almost invisible, and blood vessels are narrowed or blocked. Green arrows indicate inflammatory cells and yellow arrows indicate narrowed blood vessels. **b** Masson's trichrome stain in the lamina propria and submucosa. With the progression of OSF, fibrous degeneration of connective tissue, hyalinization of collagen gradually increased; scale bar =50 um.

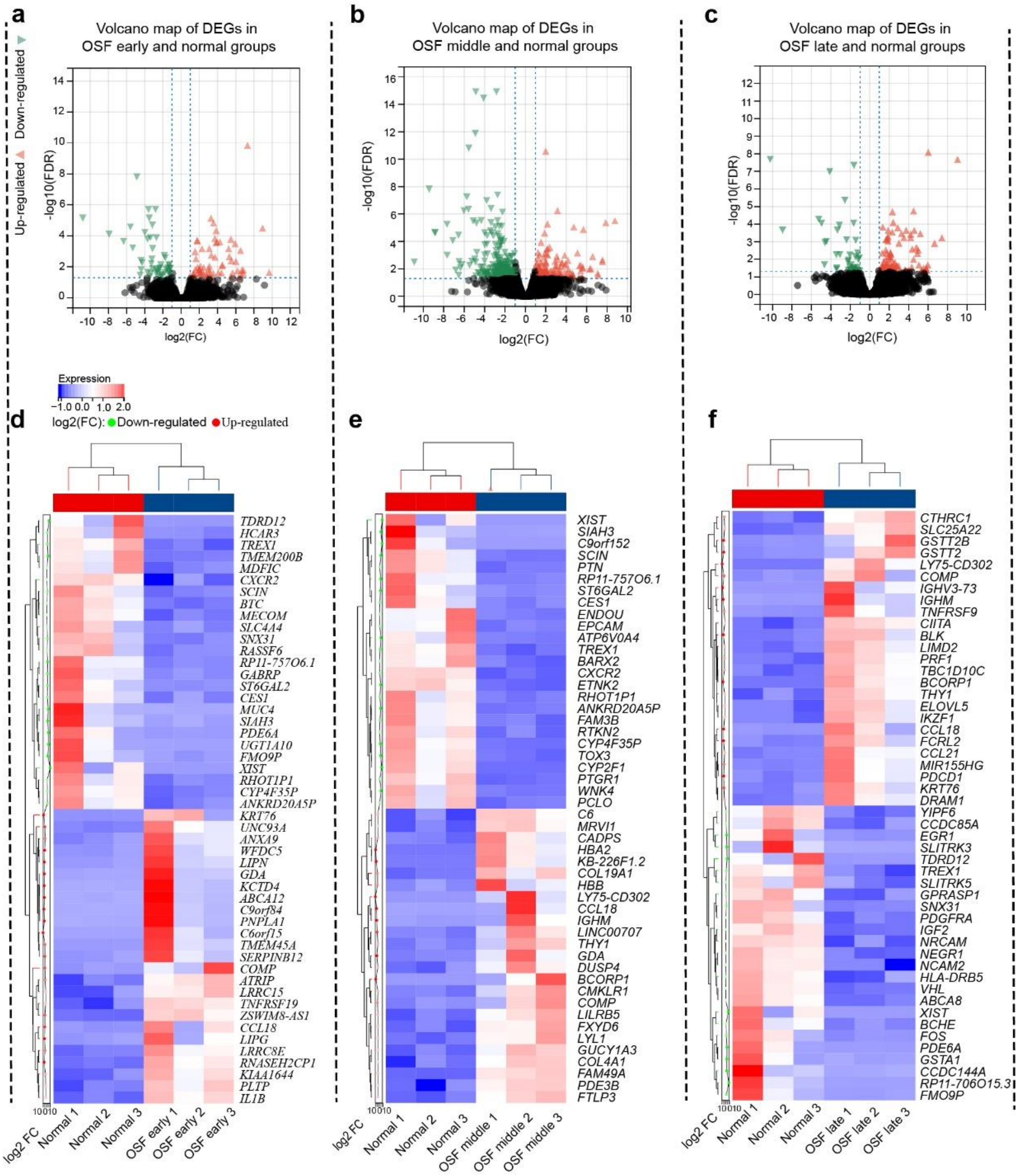


**Figure S2** **a** Volcano map of DEGs between OSF early stage and normal buccal tissues. **b** Volcano map of DEGs between OSF middle stage and normal buccal tissues. **c** Volcano map of DEGs between OSF late stage and normal buccal tissues. **d** Heatmap of the top 50 DEGs between OSF early stage and normal buccal tissues. **e** Heatmap of the top 50 DEGs between OSF middle stage and normal buccal tissues. **f** Heatmap of the top 50 DEGs between OSF late stage and normal buccal tissues. The criteria for identifying DEGS were a FDR < 0.05 and |log2(fold change (FC))| ≥ 1.

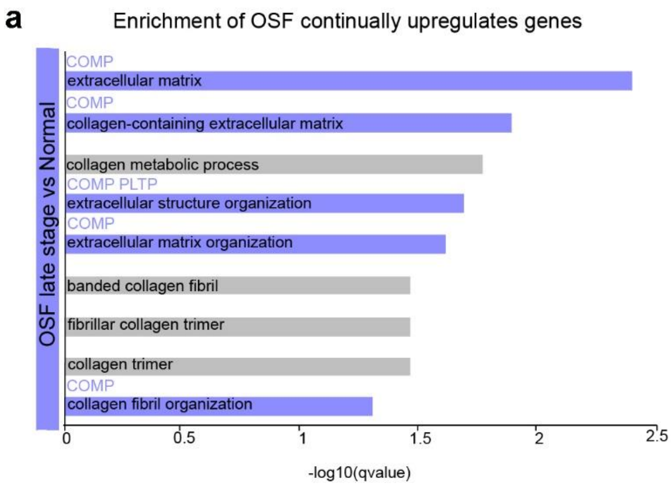


**Figure S3 a** GO analysis of differential genes in OSF late stage compared to the normal group, which contains several GO terms related to extracellular matrix and collagen metabolism. Enrichment of genes with persistent upregulation in OSF.

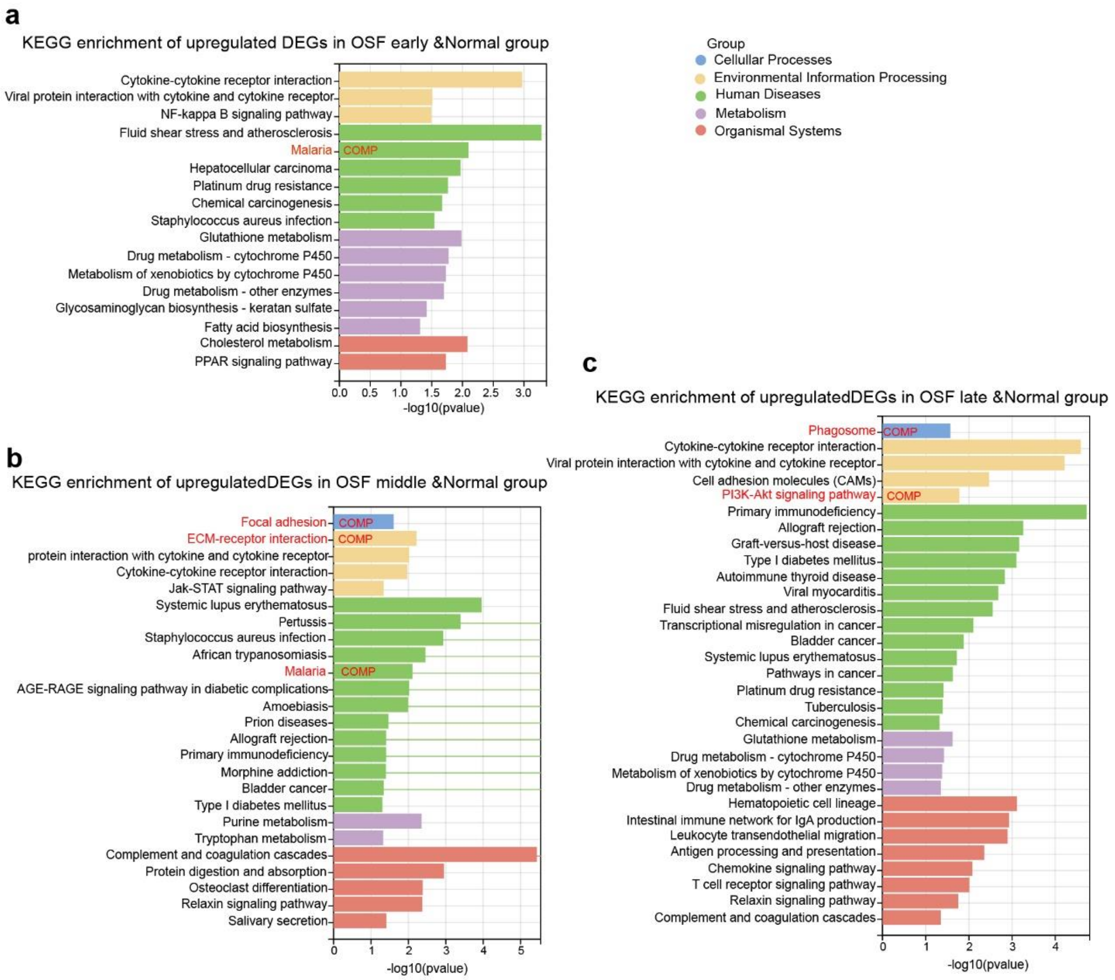


**Figure S4 a** KEGG enrichment of upregulated DEGs between OSF early stage

and normal buccal tissues. **b** KEGG enrichment of upregulated DEGs between OSF middle stage and normal buccal tissues. **c** KEGG enrichment of upregulated DEGs between OSF late stage and normal buccal tissues. Pathways in red font are COMP-related.

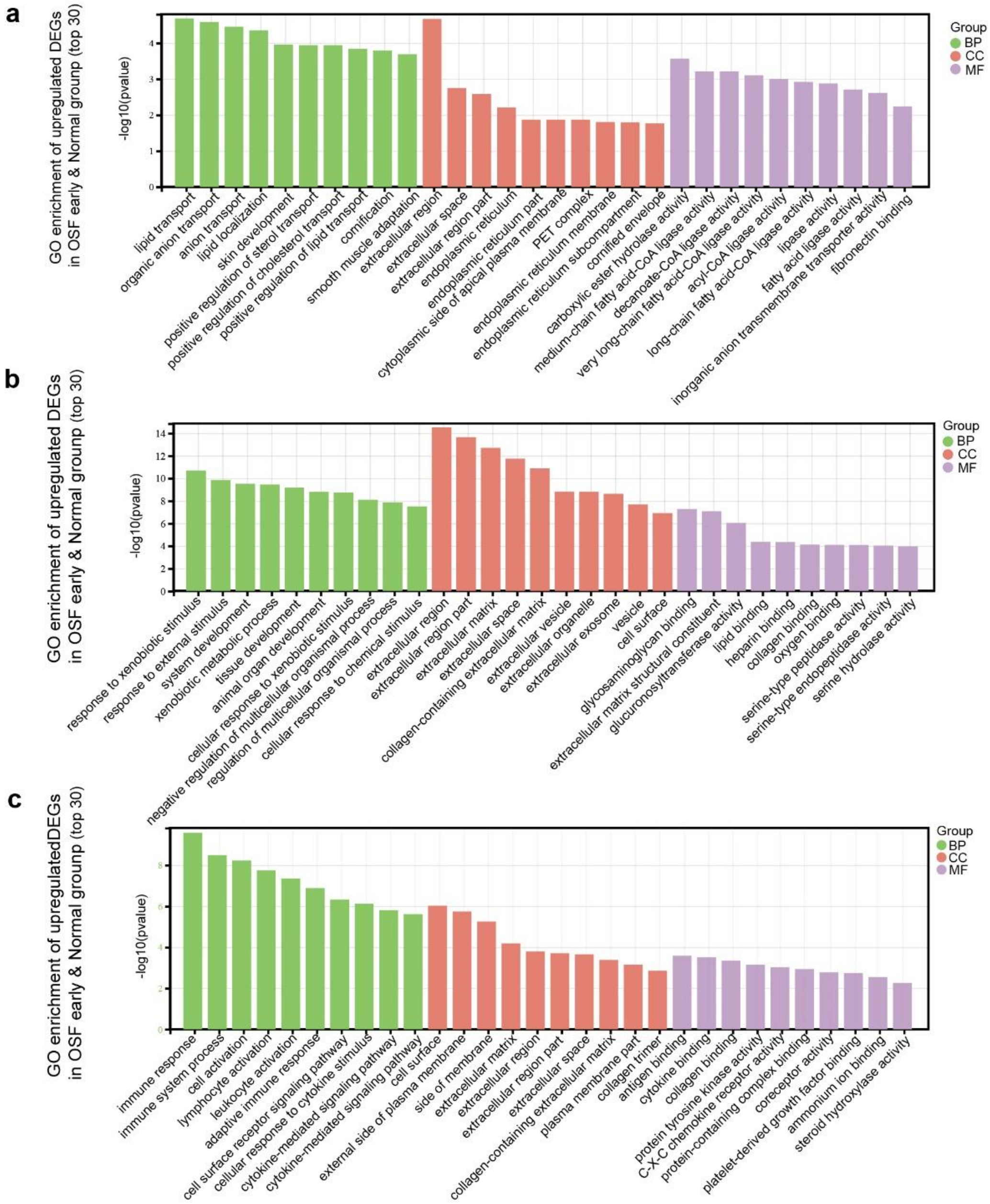


**Figure S5 a** GO enrichment of Upregulated DEGs between OSF early stage and normal buccal tissues. **b** GO enrichment of Upregulated DEGs between OSF middle stage and normal buccal tissues. **c** GO enrichment of Upregulated DEGs between OSF latelayte stage and normal buccal tissues.

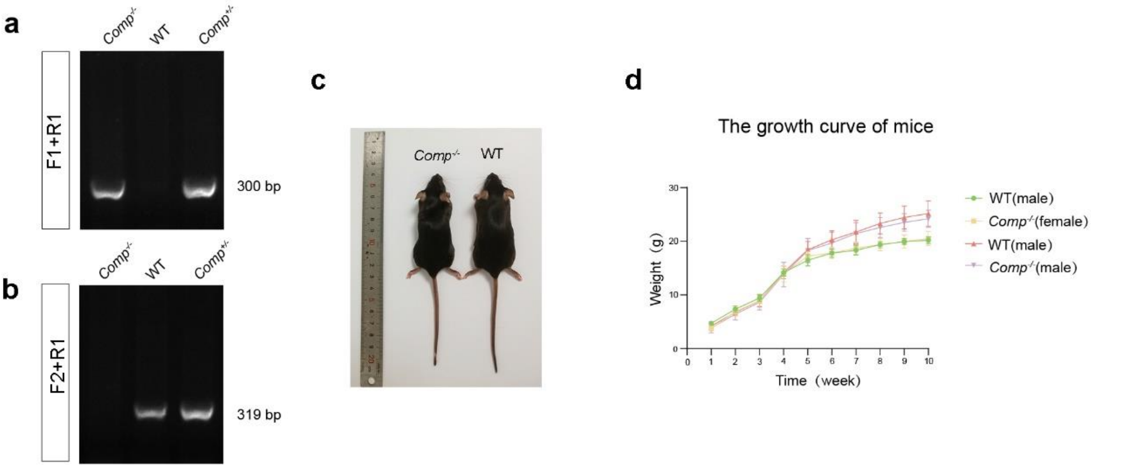


**Figure S6** Generation of *Comp*$^{-/-}$ mouse model (C57BL/6N). **a, b** Agarose gel electrophoresis analysis of gDNA isolated from mouse tail. **c** Appearance of WT mice and *Comp*$^{-/-}$ mice. **d** The growth curve of mice.

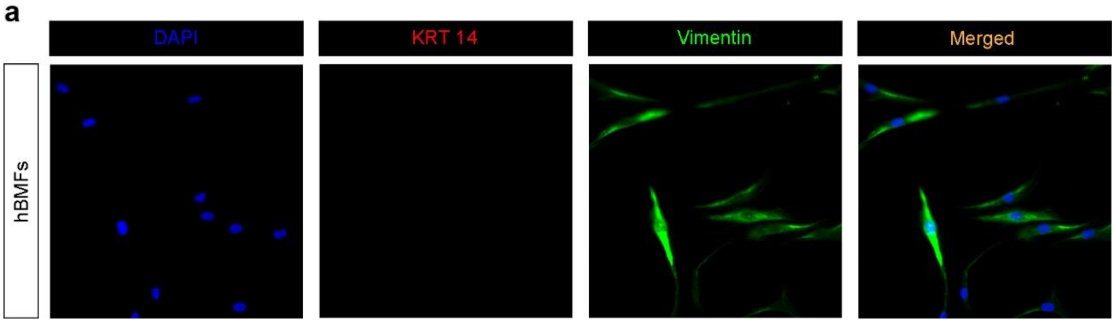


**Figure S7** Identification of hBMFs. **a** Immunofluorescence staining of KRT14 and Vimentin.

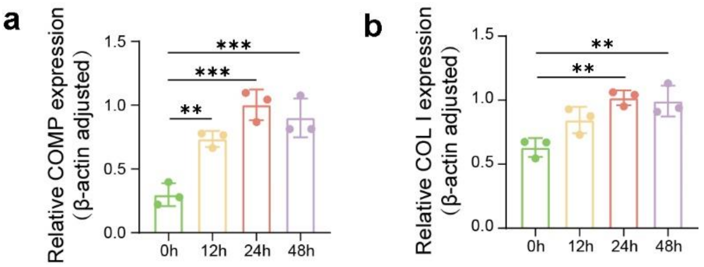


**Figure S8** Western blot analysis of collagen I, and COMP in hMBFs treated with arecoline. Data are presented as mean ± SD, ** $p < 0.01$, *** $p < 0.001$.

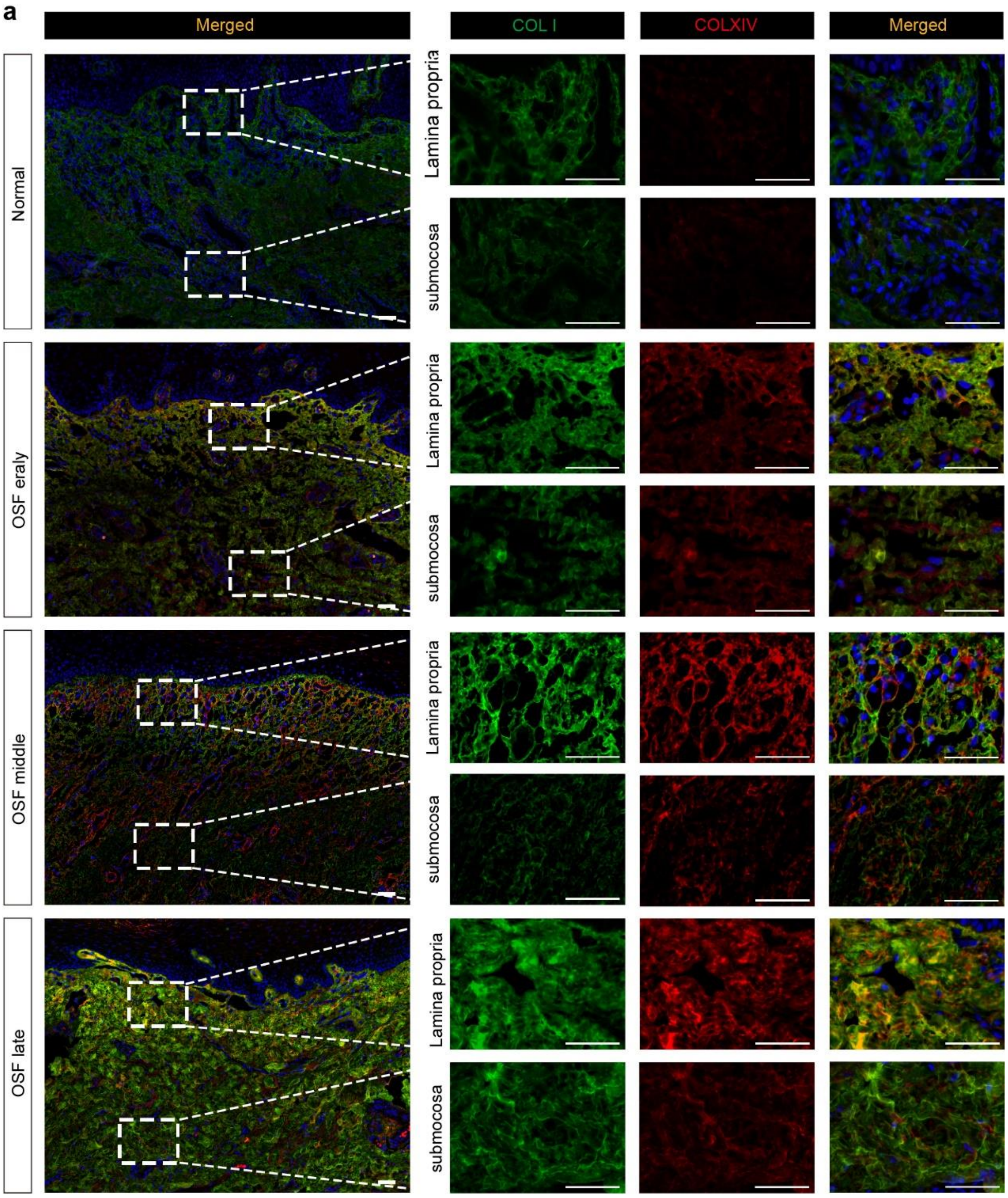


**Figure S9** Collagen XIV modifies collagen I during OSF pathogenesis. **a** Immunofluorescence co-localization of collagen XIV and collagen I in human buccal mucosal tissues, scale bar =50μm.

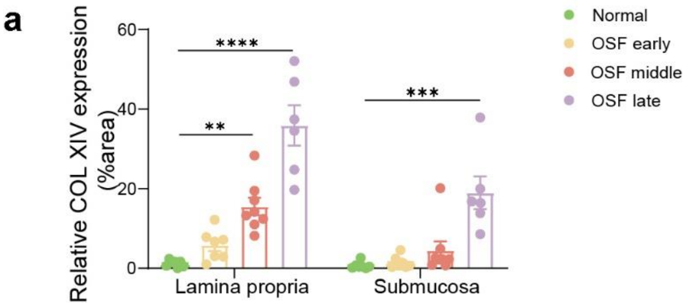

**Figure S10 a** Immunofluorescence assay of collagen XIV distribution in the lamina propria and submucosa. n =28, Data are presented as mean ± SD, **p < 0.01, ***p < 0.001, ****p < 0.0001.

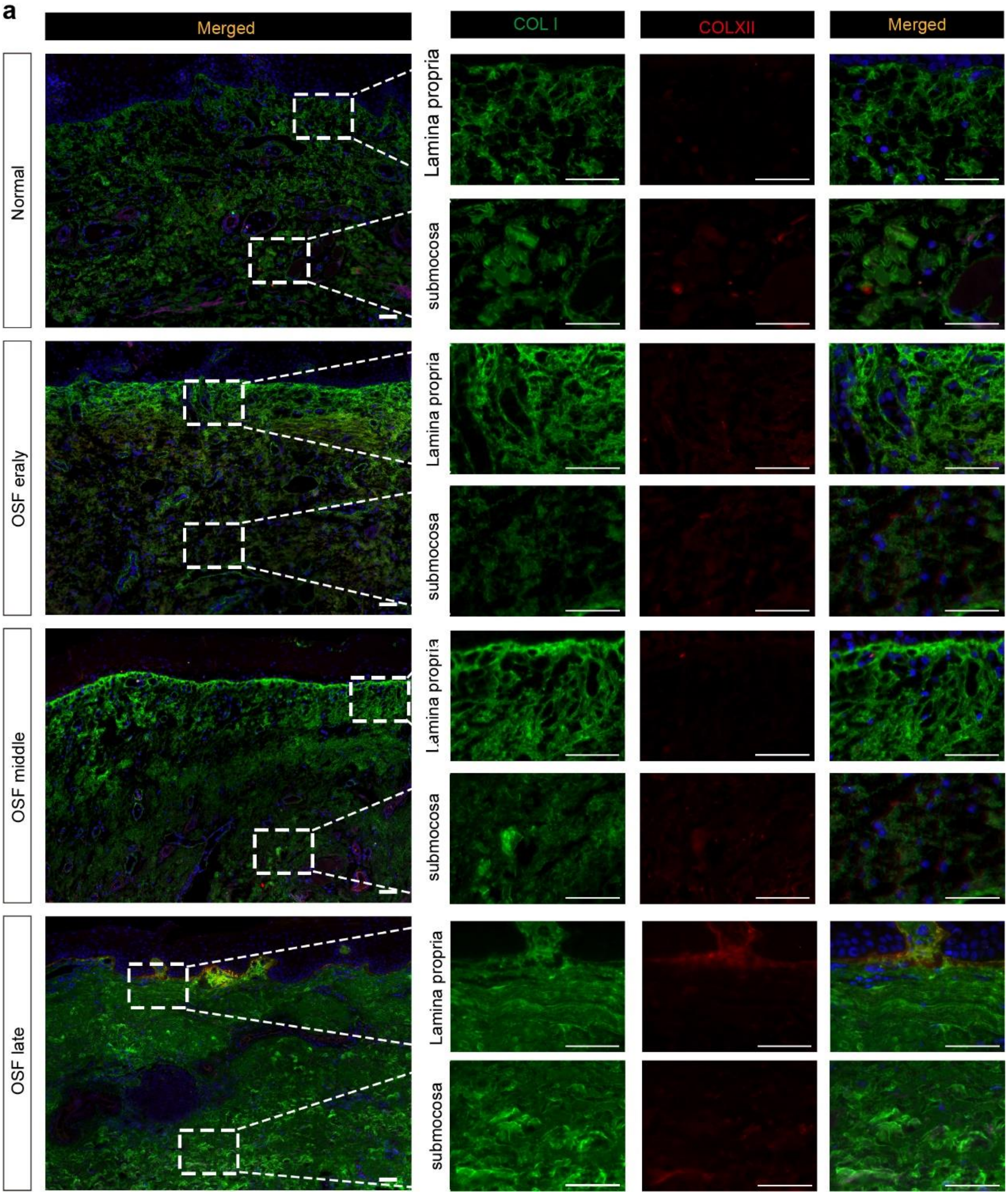


**Figure S11** Collagen XII don't modified collagen I during OSF pathogenesis. **a** Immunofluorescence co-localization of collagen XII and collagen I in human buccal mucosal tissues, scale bar =50μm.

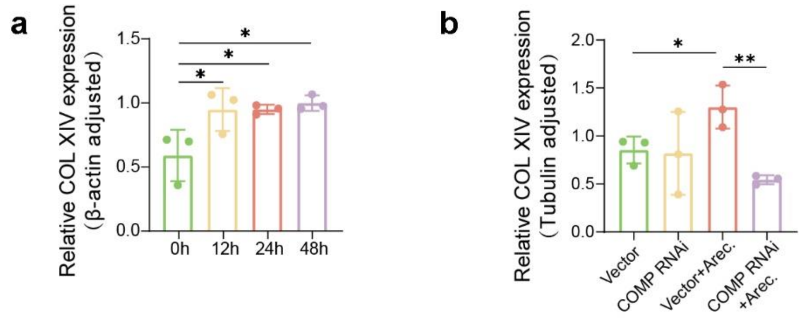


**Figure S12 a** Western blot analysis of collagen XIV in hBMFs treated with arecoline, n=12. **b** Western blot analysis of collagen XIV in shCOMP hBMFs treated with arecoline, n=12. Data are presented as mean ± SD, *p < 0.05, **p < 0.01.
